# Prefrontal rTMS modulates dynamic expectancy processes to shape subsequent pain experience

**DOI:** 10.1016/j.neurot.2026.e01107

**Published:** 2026-10-28

**Authors:** Richu Jin, Zhouan Liu, Jia Li, Yinglin Guan, Guohui Wang, Xiaoyun Li, Weiwei Peng

**Affiliations:** a Tech X Academy, Shenzhen Polytechnic University, Shenzhen, China; b School of Psychology, Shenzhen University, Shenzhen, China

**Keywords:** Pain, Expectancy, Repetitive transcranial magnetic stimulation (rTMS), Dorsolateral prefrontal cortex (DLPFC), Event-related potentials (ERPs)

## Abstract

Pain expectancy is a major determinant of pain perception, and identifying effective approaches to modulate expectancy may provide new opportunities for pain intervention. High-frequency repetitive transcranial magnetic stimulation (rTMS) targeting the dorsolateral prefrontal cortex (DLPFC) has been implicated in expectancy-related pain modulation. However, it remains unclear how DLPFC-rTMS influences distinct stages of expectancy processing and how these changes relate to subsequent pain experience. Thirty-three healthy participants completed a randomized, double-blind, sham-controlled crossover study. Participants received real or sham high-frequency DLPFC-rTMS and performed a pain expectancy paradigm before and after stimulation while EEG was recorded. The paradigm dissociated expectancy formation and maintenance under certain and uncertain predictive contexts. Behaviorally, DLPFC-rTMS reduced pain intensity and unpleasantness, accompanied by a reduction in expected pain, and mediation analysis identified an indirect effect through expected pain. At the neural level, DLPFC-rTMS increased contextual cue-evoked P2 responses relative to sham during expectancy formation but reduced anticipatory N1 and contingent negative variation (CNV) responses during expectancy maintenance. During pain experience, laser-evoked N1 responses showed a context-dependent pattern, with greater attenuation under uncertainty. Importantly, mediation analyses identified distinct stage-related indirect associations: stimulation-related changes in contextual P2 responses were linked to subsequent laser-evoked N1 responses, whereas changes in anticipatory CNV and anxiety were jointly associated with subsequent pain ratings. Together, these findings demonstrate that DLPFC-rTMS modulates dynamic expectancy processes across distinct stages of pain anticipation, with formation- and maintenance-related changes linked to different aspects of subsequent pain processing.

## Introduction

Pain is not a passive readout of nociceptive input but rather an active perceptual experience shaped by interactions among sensory, cognitive, emotional, and contextual factors [1], [2]. Among these influences, expectancy is considered one of the most powerful determinants of pain perception [3], [4], [5]. Anticipation of pain can substantially amplify or attenuate subsequent pain experience and has been implicated in a wide range of pain-related phenomena [6], [7], [8], [9], [10], [11], [12], including placebo analgesia, nocebo hyperalgesia, and predictive modulation of pain. Beyond its role in pain processing, dysfunctional expectancy has increasingly been recognized as an important contributor to clinical conditions [3], [13], [14]. Individuals with chronic pain often exhibit exaggerated threat anticipation and heightened sensitivity to uncertain predictive information [14], [15], while anxiety and depressive disorders are frequently associated with persistent negative expectancy biases [16], [17], [18]. Such maladaptive expectancy processes may further amplify pain sensitivity and contribute to symptom maintenance [13], [19]. Consequently, identifying effective approaches to modulate expectancy may provide a promising avenue for influencing pain experience and improving pain-related outcomes.

The dorsolateral prefrontal cortex (DLPFC) has emerged as a promising target for modulating expectancy-related pain processes [20], [21]. Neuroimaging studies of placebo analgesia have shown that prefrontal activity during pain anticipation is associated with subsequent reductions in pain-related responses within nociceptive brain networks [10], [22], [23]. These findings suggest that prefrontal regions may contribute to pain modulation by shaping anticipatory processing before nociceptive input occurs. Consistent with this view, the DLPFC has been implicated in predictive processing, cognitive control, and top-down regulation of pain [3], [24], [25], [26], [27], [28], [29], making it a plausible neural substrate through which expectancy influences pain experience. Extending these correlational findings, neuromodulation studies have provided evidence that high-frequency repetitive transcranial magnetic stimulation (rTMS) targeting the DLPFC can alter pain anticipation and reduce subsequent pain perception [26], [27]. However, previous studies have primarily examined expectancy in terms of overall anticipatory responses or subsequent pain outcomes [4], [7], [10], [27]. Whether DLPFC-rTMS differentially modulates the processes through which expectancy is initially formed and subsequently maintained remains unclear.

Emerging evidence suggests that pain expectancy is not a unitary anticipatory state but a dynamic process that unfolds across successive stages [30], [31], [32], [33]. Following the presentation of predictive information, individuals first encode and evaluate contextual cues to construct an expectation about a forthcoming event (expectancy formation). This expectation is then maintained throughout the anticipatory interval (expectancy maintenance) before being integrated with incoming nociceptive information during pain experience [30], [32], [34]. These stages involve partly distinct psychological operations, including contextual cue evaluation, sustained anticipatory processing, and the processing of sensory input in light of prior expectations. Accordingly, they may exhibit different neural signatures and differ in their sensitivity to DLPFC modulation. Predictive certainty may further shape these stage-dependent processes. Anticipating an event that is certain to be painful differs from anticipating one that may or may not be painful [12], [35], [36]. Uncertainty may increase demands on attentional allocation and vigilance, potentially changing how expectations are constructed and maintained and how they relate to subsequent pain responses [7], [9], [33], [37]. Nevertheless, how prefrontal neuromodulation influences expectancy formation and maintenance across different predictive contexts remains unclear.

To address these questions, the present study investigated whether DLPFC-rTMS exerts differential effects across distinct stages of expectancy processing and varying levels of predictive certainty. We combined high-frequency DLPFC-rTMS with a pain expectancy paradigm and electroencephalography (EEG) recordings to characterize the neural and behavioral consequences of prefrontal modulation. The paradigm was specifically designed to dissociate expectancy formation and maintenance processes under both certain and uncertain predictive contexts. Contextual cues were used to probe the formation of expectancy, whereas anticipatory cues preceding pain delivery were used to assess the maintenance of anticipatory states. Pain-related behavioral ratings and laser-evoked cortical responses were further examined to determine how changes in expectancy processing influence subsequent pain experience. We hypothesized that DLPFC-rTMS would alter expectancy-related processing and reduce pain perception. By distinguishing expectancy formation and maintenance within a single experimental framework, the present study moves beyond treating expectancy as a unitary anticipatory state. It further examines when prefrontal stimulation exerts its effects during anticipation and how stage-specific changes relate to subsequent neural and subjective pain responses.

## Materials and Methods

### Participants

The target sample size was determined by an *a priori* power analysis conducted using G∗Power [38], based on the effect size reported in our previous study [27] examining the effects of DLPFC-rTMS on experimental pain perception (*f* = 0.27). With α = 0.05 and a statistical power of 0.95, the analysis indicated that 32 participants were required. To account for an anticipated dropout rate of approximately 10%, we recruited 36 healthy volunteers (20 females; mean age ± standard error of mean [SEM] = 20.86 ± 0.37 years). All participants were right-handed and had normal or corrected-to-normal vision. Exclusion criteria included a history of acute or chronic pain, neurological, psychiatric, or cardiovascular disorders, current use of centrally acting medication, and any contraindications to TMS. Data from three participants were excluded because of incomplete datasets or excessive EEG artifacts, leaving 33 participants (17 females; mean age ± SEM = 20.94 ± 0.38 years) in the final analyses. All participants provided written informed consent before participation. The study was approved by the Ethics Committee of the Medical School of Shenzhen University and was conducted in accordance with the Declaration of Helsinki and relevant ethical guidelines and regulations.

### General experimental procedure

This study employed a randomized, double-blind, sham-controlled crossover design. Each participant completed two experimental sessions separated by a 7-day washout period and received real and sham DLPFC-rTMS in a randomized order. The stimulation sequence (real-first or sham-first) was determined using a computer-generated randomization sequence. The experimenter administering rTMS had access to the allocation sequence and delivered the corresponding stimulation protocol but was not involved in behavioral assessments, EEG data collection, or subsequent data analyses. Participants and the experimenter responsible for outcome assessment remained blinded to the stimulation condition throughout both sessions.

As illustrated in Fig. 1A, each session consisted of a pre-stimulation assessment, a 7.5-min rTMS intervention, and a post-stimulation assessment. During both pre- and post-stimulation assessments, participants completed a pain expectancy task while EEG was continuously recorded. The task was designed to examine expectancy formation, expectancy maintenance, and subsequent pain processing under different predictive contexts. Noxious laser stimuli were delivered to the dorsum of the left hand, and participants verbally rated pain intensity and unpleasantness using a 0–10 numerical rating scale (NRS). Before the experiment, laser intensity was individually calibrated to evoke a moderate level of pain (approximately 6 on the NRS), thereby reducing interindividual variability in perceived stimulus intensity.

**Fig. 1:**
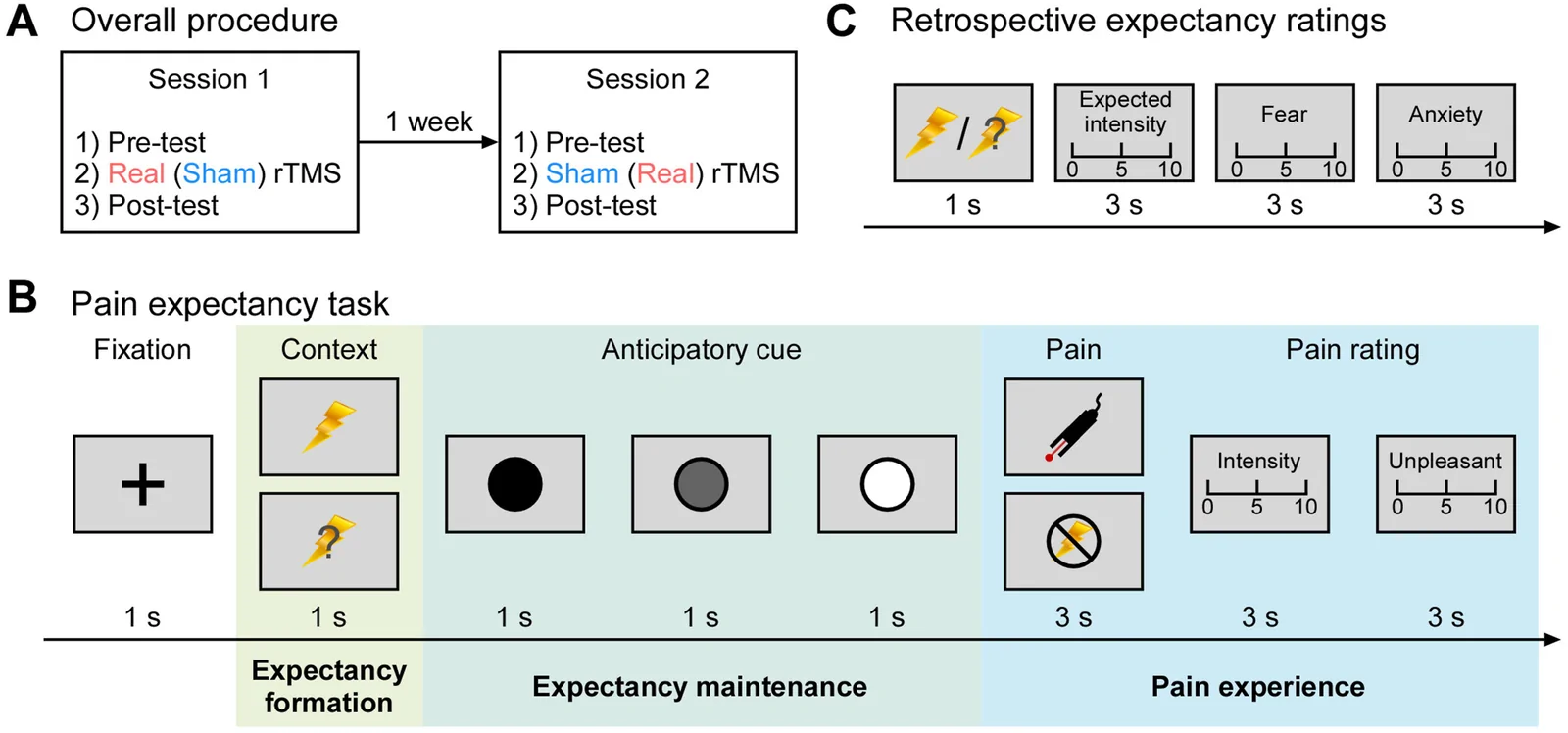
**Experimental design and pain expectancy task.*****(A) Overall experimental procedure.*** Participants completed two experimental sessions separated by a 7-day interval and received real or sham DLPFC-rTMS in a randomized, counterbalanced crossover design. Before (Pre) and after (Post) stimulation, participants performed the pain expectancy task while EEG was continuously recorded. ***(B) Pain expectancy task.*** The task comprised three successive stages: expectancy formation, expectancy maintenance, and pain experience. A contextual cue indicated either a certain (100%) or uncertain (50%) probability of pain and was used to probe expectancy formation. This was followed by a sequence of anticipatory cues (black, gray, and white dots) corresponding to expectancy maintenance. Each trial ended with either a painful laser stimulus or a no-pain outcome. Following painful trials, participants rated pain intensity and unpleasantness on 0–10 numerical rating scales (NRSs). ***(C) Retrospective expectancy ratings.*** After completing the task, participants retrospectively rated expected pain intensity, fear, and anxiety associated with each contextual cue on 0–10 NRSs.

At the beginning of the first visit, participants completed a battery of questionnaires assessing pain-related psychological characteristics, including pain sensitivity, pain-related fear, catastrophizing, vigilance, and anxiety. Questionnaire scores are summarized in Table 1.

**Table 1:** Demographic and psychometric characteristics of the participants.

	Participants (*n* = 33)
Age (years)	20.94 ± 0.38
Sex (F/M)	17/16
Pain sensitivity questionnaire	68.85 ± 2.79
Fear of pain questionnaire	94.42 ± 2.56
Pain catastrophizing scale	21.45 ± 1.77
Pain vigilance and awareness questionnaire	39.52 ± 1.17
Pain anxiety symptom scale	42.18 ± 2.71
Resting motor threshold (%)	52.03 ± 1.12
Stimulus intensity (J)	3.80 ± 0.04

**Notes:** Values are presented as mean ± standard error of the mean (SEM), unless otherwise indicated.

### Application of rTMS

Real rTMS was delivered using a MAG & More PowerMAG stimulator (MAG & More GmbH, Germany) equipped with a double PMD70-pCool figure-of-eight coil. High-frequency stimulation was applied at 10 Hz in 15 trains of 10 s duration, separated by 20-s inter-train intervals, resulting in a total of 1500 pulses over approximately 7.5 min. Stimulation targeted the left DLPFC, corresponding to the F3 location of the international 10–20 EEG system [27], [39]. Stimulation intensity was set at 80% of each participant’s resting motor threshold (RMT). RMT was determined by applying single-pulse TMS over the left primary motor cortex (M1) with the coil oriented approximately 45° relative to the midline. It was defined as the minimum stimulation intensity capable of eliciting motor-evoked potentials greater than 50 μV in at least three of five consecutive trials recorded from the relaxed first dorsal interosseous muscle [40]. Sham stimulation was delivered using a PMD70-pCool-SHAM coil that was identical to the active coil in appearance, weight, and acoustic properties. The sham coil produced similar scalp sensations while preventing effective cortical stimulation, thereby maintaining participant blinding.

### Pain expectation task

Participants completed a pain expectancy task designed to dissociate three successive stages of expectancy processing: expectancy formation, expectancy maintenance, and pain experience. The task employed a within-subject 2 (Predictive Context: certain vs. uncertain) × 2 (Outcome: pain vs. no-pain) structure. Critically, outcomes were fully determined by cue probability: certain cues always predicted pain (100% pain), whereas uncertain cues resulted in either pain or no-pain outcomes (50% probability each), yielding three realized trial types (certain pain, uncertain pain, and uncertain no-pain).

During the expectancy formation stage, participants were presented with a contextual cue that provided information about the likelihood of upcoming pain and allowed them to generate an internal prediction about the forthcoming outcome. Two contextual cues were used: a certain-pain cue (a yellow flash; one-third of trials), indicating a 100% probability of receiving a painful stimulus, and an uncertain-pain cue (a yellow flash accompanied by a question mark; two-thirds of trials), indicating a 50% probability of receiving a painful stimulus. Each contextual cue was presented for 1 s following a 1-s fixation period.

The expectancy maintenance stage began after contextual cue presentation and was designed to probe how anticipatory states were sustained over time. Participants viewed a sequence of three anticipatory cues consisting of black, gray, and white dots presented at 1-s intervals. These cues appeared at −3 s, −2 s, and −1 s relative to outcome onset, respectively, and served as a perceptual countdown that enabled participants to maintain anticipation of the upcoming outcome.

The pain experience stage corresponded to the delivery or omission of the expected stimulus. The combination of predictive context and stimulus outcome yielded three trial types: certain pain, uncertain pain, and uncertain no-pain trials. In both certain pain and uncertain pain trials, a noxious radiant-heat stimulus was delivered at the onset of the final (white) anticipatory cue. The white dot remained on the screen for an additional 3 s following stimulus onset. In uncertain no-pain trials, the white dot was replaced by a visual feedback cue indicating the absence of pain. Participants subsequently rated pain intensity and unpleasantness using a 0–10 NRS.

Noxious stimuli were generated using an infrared Nd laser (wavelength = 1.34 μm; pulse duration = 4 ms; Electronical Engineering, Italy) and delivered to the dorsum of the left hand. To minimize sensitization and habituation, the stimulation site was shifted by at least 1 cm in a random direction after each stimulus. After completing the task, participants retrospectively rated the expected pain intensity, fear, and anxiety associated with each contextual cue. The task consisted of 60 trials (20 trials per trial type) and lasted approximately 15 min.

### EEG data recordings

Continuous EEG was recorded throughout the pain expectancy task using a 32-channel Ag/AgCl electrode cap positioned according to the international 10–20 system (ANT Neuro, Enschede, The Netherlands). Signals were acquired at a sampling rate of 1000 Hz with an online bandpass filter of 0.01–100 Hz. Electrode impedances were maintained below 10 kΩ throughout the recording session. The CPz electrode served as the online reference.

### EEG data analysis

EEG data were preprocessed offline using EEGLAB [41]. Separate preprocessing pipelines were applied to expectancy-related event-related potentials (ERPs) and laser-evoked potentials (LEPs) to accommodate their different temporal characteristics. For expectancy-related ERP analyses, continuous EEG data were band-pass filtered between 0.1 and 30 Hz, with an additional 10-Hz low-pass filter applied only for visualization purposes. Epochs were extracted from −200 to 800 ms relative to cue onset and baseline-corrected using the −200 to 0 ms pre-stimulus interval. For LEP analyses, continuous EEG data were band-pass filtered between 1 and 30 Hz. Epochs were extracted from −500 to 2000 ms relative to laser stimulus onset and subsequently restricted to −200 to 1000 ms for LEP quantification. LEP epochs were baseline-corrected using the −200 to 0 ms pre-stimulus interval.

Epochs containing gross movement artifacts were initially excluded following visual inspection. Independent component analysis (ICA) was then performed to identify and remove artifactual components [42]. Independent components were classified using ICLabel, and components classified as muscle, eye, or channel noise with a classification probability ≥0.80 were flagged using the EEGLAB *pop_icaflag* function and removed. Following ICA correction, the remaining epochs were visually inspected for residual artifacts, and clearly contaminated epochs were excluded from further analyses. Trial retention after preprocessing was high, with more than 95% of trials retained across all experimental conditions. Trial rejection rates did not differ significantly between the real- and sham-rTMS sessions (expectancy-related ERPs: *p* = 0.325; LEPs: *p* = 1.00). The mean numbers of artifact-free trials retained in each experimental condition are reported in Supplementary Table S1. Following artifact correction, EEG data were re-referenced to the average of the bilateral mastoids.

#### Expectancy formation: contextual cue-evoked responses

To characterize neural processes involved in expectancy formation, ERPs elicited by the contextual cues were analyzed. For each participant, ERP waveforms were averaged separately for each experimental condition (2 predictive contexts × 2 rTMS types × 2 time points), resulting in eight condition-specific waveforms. Group-level waveforms and scalp topographies were obtained by averaging across participants. Based on grand-average scalp distributions and previous expectancy studies [43], the P2 and P3 components were quantified at the stimulation target region (F3). Mean amplitudes were measured within 160–200 ms (P2) and 400–700 ms (P3) following contextual cue onset.

#### Expectancy maintenance: anticipatory cue-evoked responses

To characterize neural processes involved in expectancy maintenance, ERPs elicited by the final anticipatory cue immediately preceding outcome delivery were analyzed. Using the same averaging procedure described above, N1 and contingent negative variation (CNV) components were quantified at F3 according to their maximal scalp distributions and previous literature [7], [44]. Mean amplitudes were measured within 100–160 ms (N1) and 400–700 ms (CNV) following anticipatory cue onset.

#### Pain experience: laser-evoked responses

To characterize neural responses during pain experience, LEPs elicited by laser stimulation were analyzed separately for certain and uncertain pain trials. Single-trial LEPs were averaged within each experimental condition (2 predictive contexts × 2 rTMS types × 2 time points), yielding eight averaged waveforms per participant. Group-level waveforms and scalp topographies were then obtained by averaging across participants. The vertex N2 and P2 components were quantified as mean amplitudes at Cz within 200–260 ms and 300–400 ms following stimulus onset, respectively. In addition, the N1 component, which reflects early nociceptive processing within the sensorimotor system, was quantified at the contralateral sensorimotor electrodes (C4, CP2, and CP6) within 190–230 ms and referenced to Fz [45].

#### Statistical analysis

All statistical analyses were performed using IBM SPSS Statistics (version 22; IBM Corp., Armonk, NY, USA). To evaluate changes associated with real versus sham DLPFC-rTMS relative to each session’s pre-stimulation baseline, Post–Pre difference scores were calculated for each behavioral and electrophysiological outcome. These scores were analyzed using two-way repeated-measures analyses of variance (ANOVAs), with rTMS Type (real vs. sham) and Predictive Context (certain vs. uncertain) as within-subject factors. Significant interactions were followed by Bonferroni-adjusted pairwise comparisons. Partial eta-squared (η_p_^2^) was reported as a measure of effect size. To account for multiple comparisons, the Benjamini–Hochberg false discovery rate (FDR) procedure [46] was applied to omnibus tests within theoretically defined behavioral and electrophysiological outcome domains. Both uncorrected and FDR-adjusted *p* values are reported.

Additional sensitivity analyses were conducted to evaluate the robustness of the primary change-score analyses. Baseline comparability between the real- and sham-rTMS sessions was examined using pre-stimulation measurements, and baseline-adjusted analyses were performed where appropriate. Potential stimulation-sequence effects were assessed by incorporating Stimulation Sequence (real-first vs. sham-first) as a between-subject factor in mixed-design ANOVAs of the change scores. In addition, the original pre- and post-stimulation measurements were analyzed using three-way repeated-measures ANOVAs with rTMS Type, Predictive Context, and Time (pre vs. post) as within-subject factors. Full details and results of these analyses are provided in the Supplementary Table S2–S4.

To examine the statistical associations between rTMS-related changes in expectancy-related measures and subsequent pain-related outcomes, mediation analyses were conducted using the MEMORE macro for SPSS [47]. Indirect effects were estimated using bias-corrected bootstrap procedures with 5000 resamples and were considered statistically supported when their 95% confidence intervals (CIs) did not include zero. As an exploratory assessment of multiplicity across the three theoretically specified indirect effects, empirical two-sided bootstrap tail probabilities were calculated from the bootstrap estimates and adjusted using the Benjamini–Hochberg FDR procedure.

## Results

### Blinding efficacy and tolerability

An exact McNemar’s test revealed no significant difference in participants’ judgments regarding whether they had received real or sham stimulation (*p* = 1.00), providing no evidence that participants could distinguish between the two stimulation conditions. As summarized in Table 2, the reported adverse events were comparable between real- and sham-rTMS sessions (all *ps* > 0.34), indicating that both stimulation conditions were generally well tolerated.

**Table 2:** Reported adverse effects following real and sham DLPFC-rTMS.

	Real	Sham	McNemar test (*p*)
Headache (No/Yes)	28/5	29/4	1.00
Neck pain (No/Yes)	32/1	33/0	1.00
Scalp pain (No/Yes)	28/5	29/4	1.00
Tingling (No/Yes)	28/5	26/7	0.50
Seizures (No/Yes)	33/0	33/0	1.00
Hearing impairment (No/Yes)	33/0	32/1	1.00
Cognitive impairment (No/Yes)	33/0	33/0	1.00
Trouble concentrating (No/Yes)	20/13	24/9	0.34
Acute mood changes (No/Yes)	31/2	32/1	1.00

**Notes:** Values are presented as the number of participants reporting absence/presence of each adverse effect. *P* values were obtained using McNemar’s tests comparing real and sham stimulation sessions.

### DLPFC-rTMS reduces pain expectancy and pain perception

Pre-stimulation behavioral measures were generally comparable between the real- and sham-rTMS sessions (Supplementary Table S2). Fig. 2A–E illustrates changes in expectancy-related ratings (expected pain, fear, and anxiety) and pain-related ratings (pain intensity and unpleasantness) following real and sham DLPFC-rTMS. The corresponding stimulation-related effects are summarized in Table 3.

**Fig. 2:**
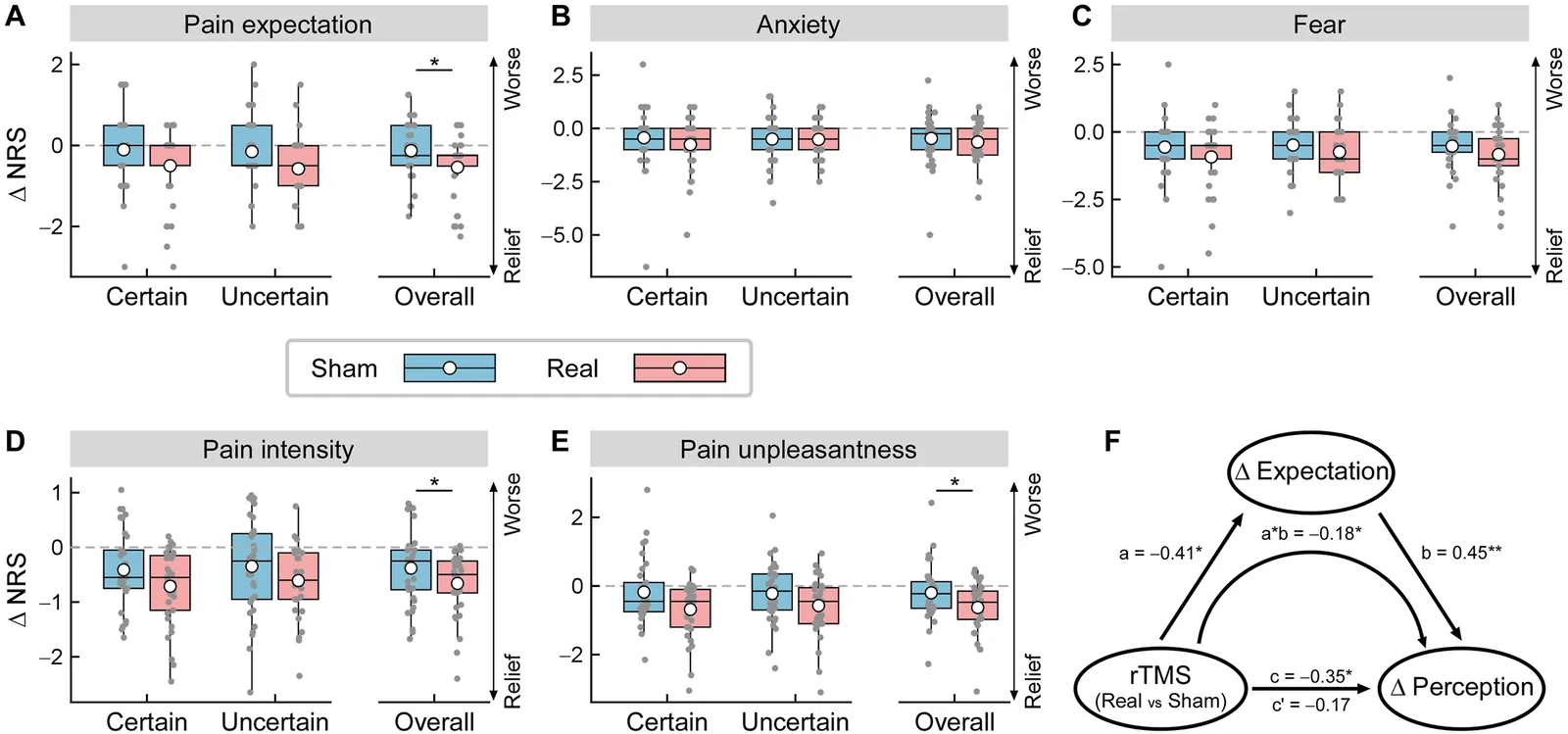
**Effects of DLPFC-rTMS on expectancy- and pain-related behavioral responses.*****(A–E) Post–Pre changes in behavioral ratings following real (red) and sham (blue) DLPFC-rTMS under certain and uncertain predictive contexts.*** Expectancy-related ratings included expected pain, anxiety, and fear, whereas pain-related ratings included pain intensity and unpleasantness. Real DLPFC-rTMS induced greater reductions in expected pain, pain intensity, and pain unpleasantness compared with sham stimulation. Box plots show the interquartile range and median (black line), with means indicated by white circles and individual participant values overlaid. ***(F) Within-participant mediation analysis examining the indirect association between DLPFC-rTMS and pain perception through expected pain.*** DLPFC-rTMS (1 = real, −1 = sham) was entered as the independent variable, expected pain as the mediator, and perceived pain (mean change in pain intensity and unpleasantness) as the dependent variable. Path coefficients are shown for the mediation model. ∗: *p* < 0.05; ∗∗: *p* < 0.01.

**Table 3:** Statistical results for the effects of DLPFC-rTMS on behavioral and neural responses.

Outcome	rTMS type			Context			rTMS type × context		
	*F*_*1, 32*_	*p*	*p*_*FDR*_	*F*_*1, 32*_	*p*	*p*_*FDR*_	*F*_*1, 32*_	*p*	*p*_*FDR*_
*Expectancy-related behavioral measures*									
Expected pain	**5.46**	**0.026**	**0.078**	0.31	0.581	1.000	0.02	0.894	0.894
Fear	2.76	0.107	0.161	0.002	0.966	1.000	0.04	0.835	0.894
Anxiety	1.36	0.253	0.253	<0.001	1.000	1.000	0.12	0.731	0.894
*Pain perception–related behavioral measures*									
Pain intensity	**4.22**	**0.048**	**0.048**	1.34	0.256	0.512	0.03	0.862	0.862
Unpleasantness	**5.46**	**0.026**	**0.048**	0.30	0.586	0.586	0.42	0.521	0.862
*Expectancy-related ERP measures*									
Contextual P2	**8.40**	**0.007**	**0.014**	1.33	0.257	0.723	**5.22**	**0.029**	**0.116**
Contextual P3	0.18	0.678	0.678	0.01	0.931	0.931	0.26	0.613	0.613
Anticipatory N1	**9.60**	**0.004**	**0.014**	0.38	0.542	0.723	1.74	0.196	0.392
CNV	**6.94**	**0.013**	**0.017**	0.81	0.376	0.723	0.56	0.458	0.611
*Pain perception-related ERP measures*									
N2–P2 complex	0.06	0.808	0.808	1.16	0.291	0.407	0.37	0.549	0.549
Laser-evoked N1	1.20	0.281	0.562	0.71	0.407	0.407	**4.69**	**0.038**	**0.076**

**Notes:** Statistical analyses were performed using two-way repeated-measures ANOVAs on Post–Pre change scores, with rTMS Type (real vs. sham) and Predictive Context (certain vs. uncertain) as within-subject factors. *p*_*FDR*_ values were obtained using the Benjamini–Hochberg false discovery rate procedure within theoretically defined outcome domains. Bold values indicate effects significant at the uncorrected level; bold *p*_*FDR*_ values indicate effects that remained significant after FDR correction.

A significant main effect of rTMS Type was observed for expected pain (*F*_*1, 32*_ = 5.46, *p* = 0.026, *p*_*FDR*_ = 0.078, η_p_^2^ = 0.15; Fig. 2A, reflecting a greater reduction following real than sham stimulation, although this effect did not survive FDR correction. No significant effects of rTMS Type, Predictive Context, or their interaction were observed for fear or anxiety ratings (all *ps* > 0.05; Fig. 2B and C).

For pain perception, significant main effects of rTMS Type were observed for both pain intensity (*F*_*1, 32*_ = 4.22, *p* = 0.048, *p*_*FDR*_ = 0.048, η_p_^2^ = 0.12; Fig. 2D) and pain unpleasantness (*F*_*1,32*_ = 5.46, *p* = 0.026, *p*_*FDR*_ = 0.048, η_p_^2^ = 0.15; Fig. 2E). Relative to sham stimulation, real DLPFC-rTMS produced greater reductions in both measures. Neither the main effect of Predictive Context nor the rTMS Type × Predictive Context interaction was significant for either outcome. Hence, DLPFC-rTMS reduced subsequent pain intensity and unpleasantness across predictive contexts and was also associated with a reduction in expected pain.

To examine whether changes in expected pain were statistically associated with the effects of DLPFC-rTMS on subsequent pain perception, we conducted a within-participant mediation analysis across predictive contexts. This analysis revealed a significant indirect effect through expected pain ratings (indirect effect = −0.18, SE = 0.11, 95% CI = [−0.48, −0.03], *p*_*FDR*_ = 0.014; Fig. 2F). This statistical indirect effect suggests that rTMS-related changes in expected pain were associated with subsequent changes in pain perception.

Additional sensitivity analyses generally supported the primary findings. Analyses of the original pre- and post-stimulation measurements yielded a pattern of results consistent with the primary change-score analyses (Supplementary Table S3). Analyses incorporating stimulation sequence as a between-subject factor also showed that the principal pattern of rTMS-related effects was largely preserved. One exception was a significant rTMS Type × Predictive Context × Stimulation Sequence interaction for expected pain (*p* = 0.019), indicating that the context-dependent pattern of expected-pain changes differed according to stimulation sequence. Full results are provided in Supplementary Table S4 and Fig. S1.

### DLPFC-rTMS differentially modulates expectancy formation and maintenance

To examine the effects of DLPFC-rTMS at distinct stages of expectancy processing, we separately analyzed contextual cue–evoked responses during expectancy formation and final anticipatory cue-evoked responses during expectancy maintenance. Statistical results for these expectancy-related ERP measures are provided in Table 3. Pre-stimulation values were generally comparable between the real- and sham-rTMS sessions, except for anticipatory N1 amplitudes (*p* = 0.034). Importantly, the effect of rTMS Type on anticipatory N1 remained significant after adjustment for baseline amplitudes (*p* = 0.019; Supplementary Table S2).

#### Expectancy formation

Grand-average ERP waveforms and scalp topographies elicited by contextual cues are shown in Fig. 3A. Contextual cue-evoked P2 and P3 responses showed prominent prefrontal and parietal distributions, respectively. As shown in Fig. 3B and Table 3, P2 amplitudes exhibited a significant main effect of rTMS Type (*F*_*1, 32*_ = 8.40, *p* = 0.007, *p*_*FDR*_ = 0.014, η_p_^2^ = 0.21). Relative to sham stimulation, real DLPFC-rTMS attenuated the decline in contextual cue–evoked P2 amplitudes from pre-to post-stimulation.

**Fig. 3:**
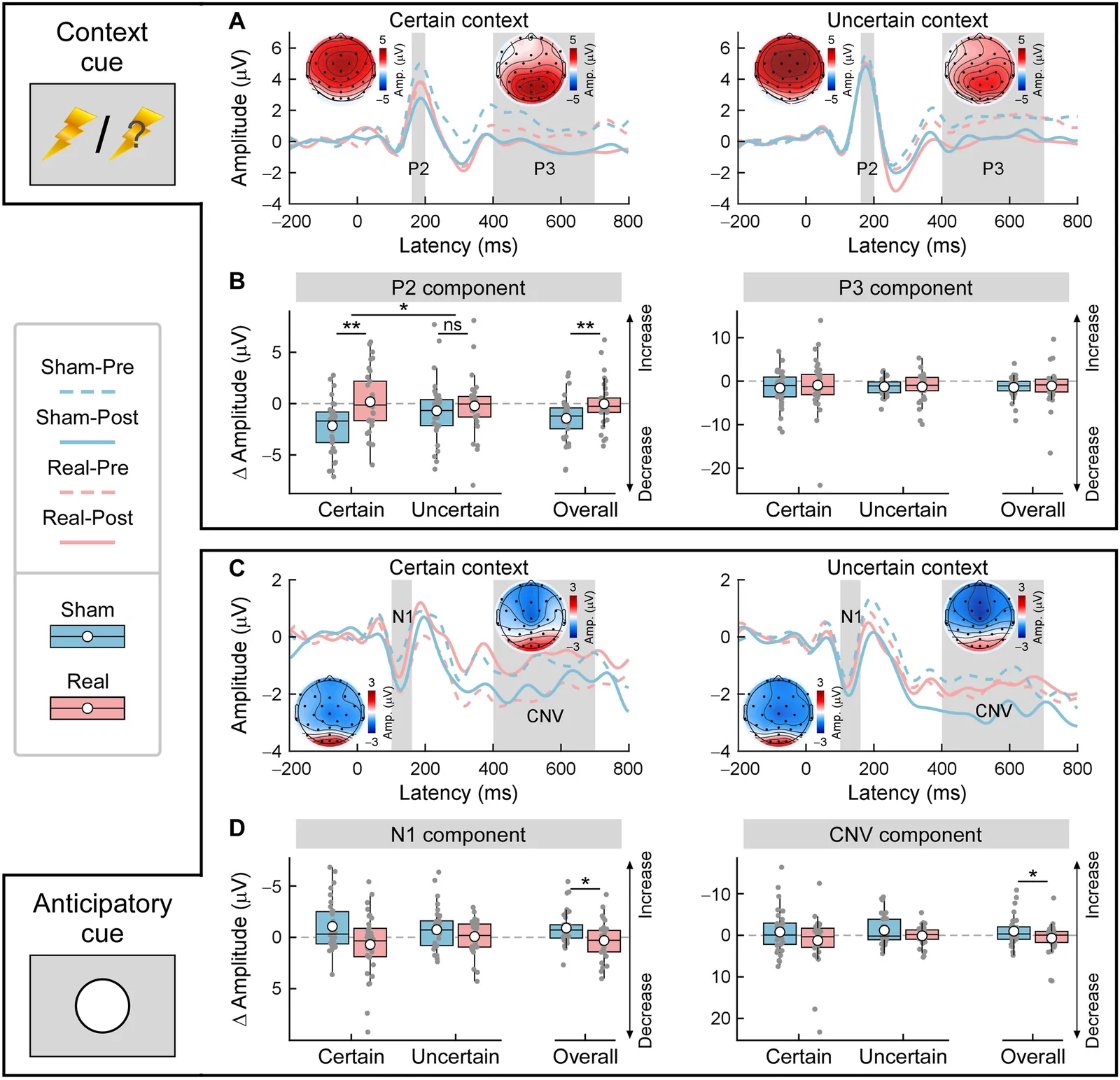
**Effects of DLPFC-rTMS on cue-evoked cortical responses during expectancy formation and maintenance.*****(A) Contextual cue-evoked ERPs during expectancy formation.*** Grand-average waveforms show ERPs elicited by contextual cues indicating certain (100%) or uncertain (50%) probability of upcoming pain. Scalp topographies illustrate the distributions of the P2 and P3 components. Gray shaded areas indicate the time windows used for component quantification. Red and blue lines denote real and sham DLPFC-rTMS, respectively; dashed and solid lines indicate pre- and post-stimulation measurements. ***(B) Changes in contextual cue–evoked responses.*** Post–Pre changes in P2 and P3 amplitudes following real and sham stimulation under certain and uncertain predictive contexts. Relative to sham stimulation, real DLPFC-rTMS attenuated the pre-to-post decline in P2 amplitude, with a more pronounced difference under the certain context, whereas no significant stimulation-related effects were observed for P3. Box plots show the interquartile range and median (black line), with means indicated by white circles and individual participant values overlaid. ***(C) Anticipatory cue-evoked ERPs during expectancy maintenance.*** Grand-average waveforms show ERPs elicited by the final anticipatory cue preceding outcome delivery. Scalp topographies illustrate the distributions of anticipatory N1 and contingent negative variation (CNV), with gray shaded areas indicating the quantification windows. ERPs were quantified at F3, corresponding to the left DLPFC stimulation site. Color and line conventions are identical to panel (A). ***(D) Changes in anticipatory cue–evoked responses.*** Post–Pre changes in N1 and CNV amplitudes following real and sham stimulation under certain and uncertain predictive contexts. Relative to sham stimulation, real DLPFC-rTMS reduced both anticipatory N1 and CNV amplitudes across contexts, indicating modulation of neural responses during expectancy maintenance. ns: *p* > 0.05; ∗: *p* < 0.05; ∗∗: *p* < 0.01.

A rTMS Type × Predictive Context interaction was also observed in the uncorrected analysis (*F*_*1, 32*_ = 5.22, *p* = 0.029, *p*_*FDR*_ = 0.116, η_p_^2^ = 0.14), although this interaction did not survive FDR correction. Post hoc comparisons showed a significant real-versus-sham difference under the certain context (*p* = 0.001), but not under the uncertain context (*p* = 0.445). No significant stimulation-related effects were observed for contextual P3 amplitudes. Hence, DLPFC-rTMS relatively increased contextual cue-evoked P2 responses during expectancy formation, particularly under certain predictive contexts.

#### Expectancy maintenance

Grand-average ERP waveforms and scalp topographies elicited by the final anticipatory cue are shown in Fig. 3C. Both anticipatory N1 and CNV responses showed prominent prefrontal distributions. As illustrated in Fig. 3D and summarized in Table 3, significant main effects of rTMS Type were observed for anticipatory N1 (*F*_*1, 32*_ = 9.60, *p* = 0.004, *p*_*FDR*_ = 0.014, η_p_^2^ = 0.23) and CNV amplitudes (*F*_*1, 32*_ = 6.94, *p* = 0.013, *p*_*FDR*_ = 0.017, η_p_^2^ = 0.18). Relative to sham stimulation, real DLPFC-rTMS reduced both anticipatory N1 and CNV amplitudes. No significant rTMS Type × Predictive Context interactions were observed. Hence, DLPFC-rTMS decreased anticipatory N1 and CNV responses during expectancy maintenance across predictive contexts.

### Context-dependent changes in laser-evoked N1 responses following DLPFC-rTMS

Grand-average laser-evoked potentials and scalp topographies are shown in Fig. 4A. The N2 and P2 components exhibited prominent vertex distributions, whereas the earlier N1 component showed a contralateral centroparietal distribution relative to stimulation of the left hand. The corresponding stimulation-related effects on laser-evoked responses are reported in Table 3.

**Fig. 4:**
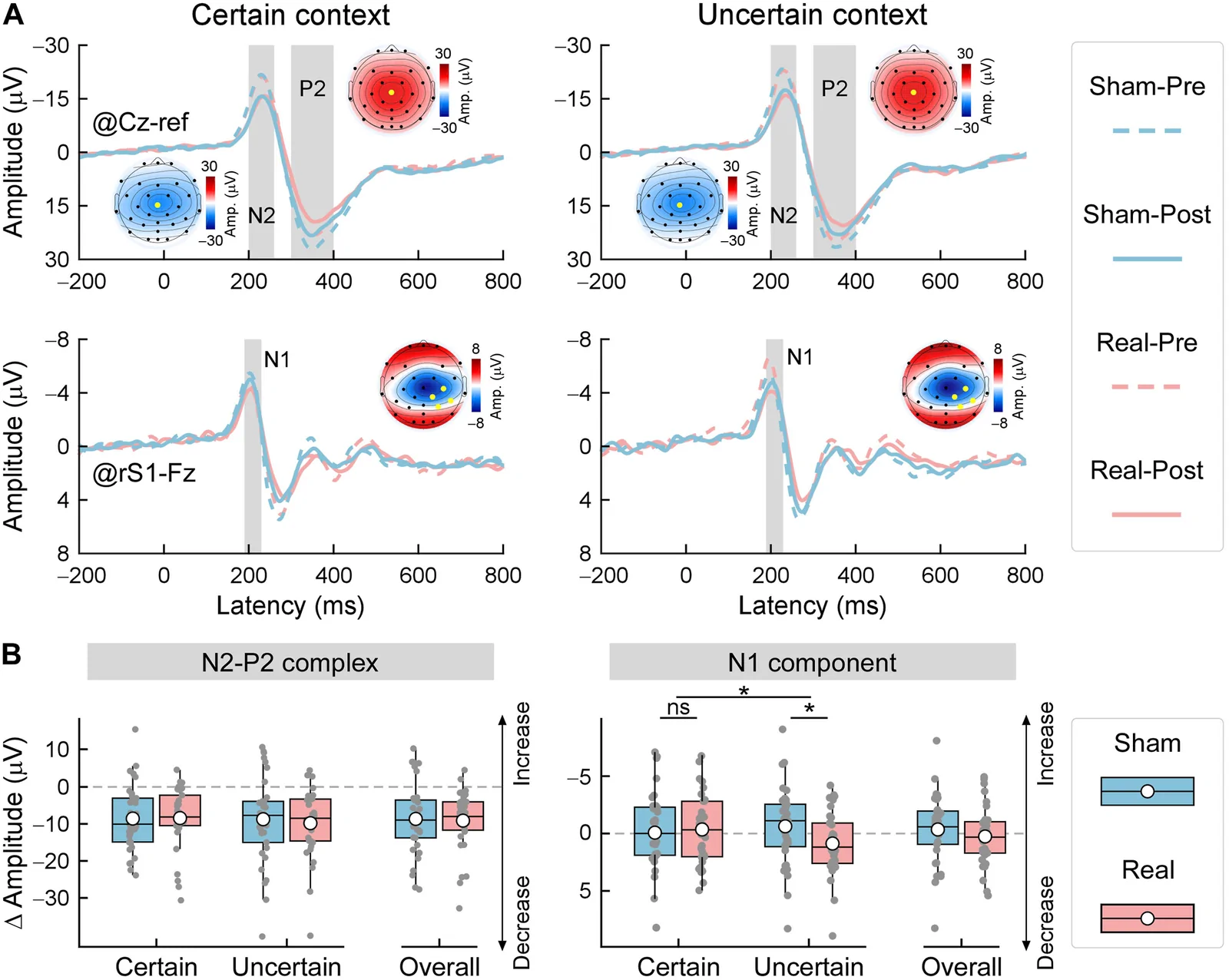
**Effects of DLPFC-rTMS on pain-evoked cortical responses.*****(A) Laser-evoked potentials (LEPs).*** Grand-average waveforms show LEPs elicited by painful laser stimulation under certain and uncertain predictive contexts. The N2–P2 complex was quantified at Cz, whereas the N1 component was quantified over right centroparietal electrodes (C4, CP2, and CP6), contralateral to stimulation of the left hand, using an Fz reference. Scalp topographies illustrate the spatial distributions of the N2, P2, and N1 components. Gray shaded areas indicate the time windows used for component quantification. Red and blue lines denote real and sham DLPFC-rTMS, respectively; dashed and solid lines indicate pre- and post-stimulation measurements. ***(B) Changes in pain-evoked responses.*** Post–Pre changes in N2–P2 and N1 amplitudes following real and sham stimulation under certain and uncertain predictive contexts. Relative to sham stimulation, real DLPFC-rTMS produced a greater reduction in laser-evoked N1 amplitude under the uncertain context, whereas no significant stimulation-related effects were observed for the N2–P2 complex. Raincloud plots show the data distributions, including box plots (interquartile range and median), means (white circles), and individual participant values. ns: *p* > 0.05; ∗: *p* < 0.05.

As shown in Fig. 4B and Table 3, no significant main effects or interactions involving rTMS Type were observed for the N2–P2 complex. For laser-evoked N1 amplitudes, the uncorrected analysis revealed a rTMS Type × Predictive Context interaction (*F*_*1, 32*_ = 4.69, *p* = 0.038, *p*_*FDR*_ = 0.076, η_p_^2^ = 0.13), although this interaction did not survive FDR correction. Post hoc comparisons showed a greater reduction in N1 amplitude following real than sham stimulation under the uncertain context (*p* = 0.033), whereas no significant difference was observed under the certain context (*p* = 0.699). These results indicate a context-dependent pattern of DLPFC-rTMS effects on early laser-evoked N1 responses, with greater attenuation under uncertain than certain predictive contexts; however, this interaction was less robust after correction for multiple comparisons.

### Distinct expectancy pathways link prefrontal modulation to pain outcomes

The behavioral mediation analysis identified an indirect effect of DLPFC-rTMS on pain perception through changes in expected pain. We next examined whether neural responses during expectancy formation and maintenance showed indirect associations with subsequent pain-related outcomes.

For expectancy formation, mediation analysis identified a significant indirect effect of DLPFC-rTMS on laser-evoked N1 amplitudes through contextual cue-evoked P2 responses (indirect effect = −0.40, SE = 0.28, 95% CI [−1.18, −0.01], *p*_*FDR*_ = 0.042; Fig. 5A). This effect indicates that rTMS-related changes in contextual cue-evoked P2 responses were statistically linked to subsequent changes in early laser-evoked N1 responses.

**Fig. 5:**
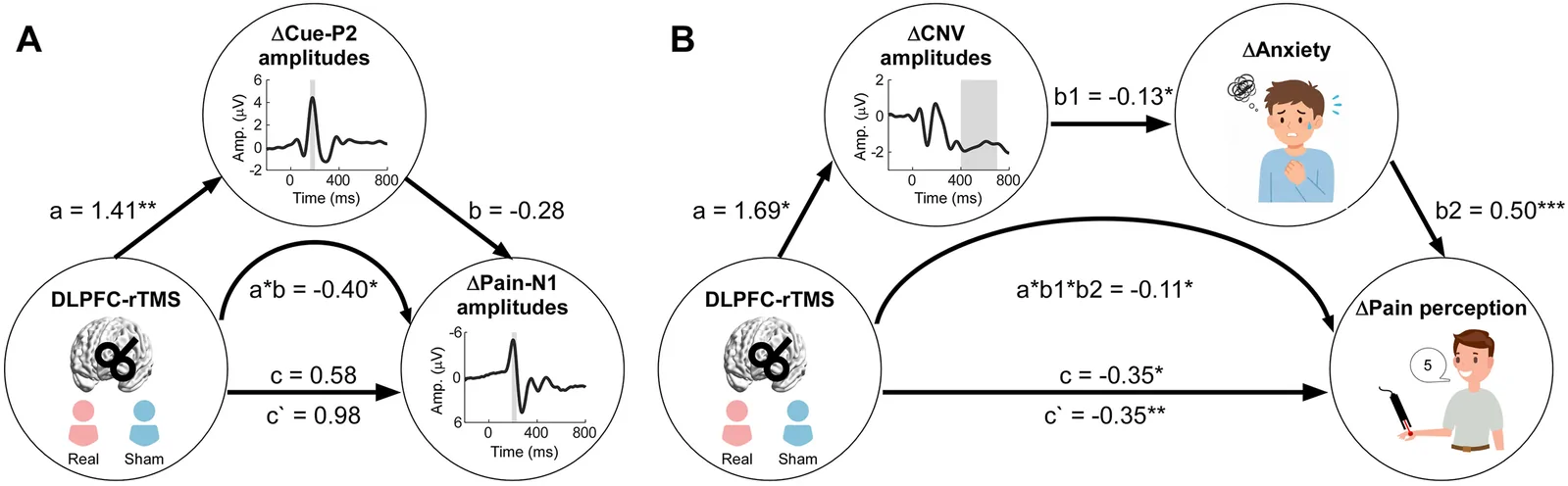
**Mediation models linking DLPFC-rTMS, expectancy-related processes, and pain outcomes.*****(A) Expectancy formation.*** Within-participant mediation analysis examining the indirect association between DLPFC-rTMS and laser-evoked N1 responses through contextual cue-evoked P2 responses. DLPFC-rTMS (1 = real, −1 = sham) was entered as the independent variable, changes in contextual cue-evoked P2 amplitude as the mediator, and changes in laser-evoked N1 amplitude as the dependent variable. The significant indirect effect indicates a statistical association linking stimulation-related changes in contextual cue processing to subsequent early pain-evoked neural responses. ***(B) Expectancy maintenance.*** Serial mediation analysis examining the indirect association between DLPFC-rTMS and pain perception through anticipatory CNV responses and anxiety ratings. DLPFC-rTMS (1 = real, −1 = sham) was entered as the independent variable, changes in CNV amplitude and anxiety ratings as sequential mediators, and pain perception (mean change in pain intensity and unpleasantness ratings) as the dependent variable. The significant serial indirect effect links stimulation-related changes in anticipatory neural and affective responses to subsequent subjective pain perception. Path coefficients are shown for each model. ∗: *p* < 0.05; ∗∗: *p* < 0.01; ∗∗∗: *p* < 0.001.

For expectancy maintenance, serial mediation analysis identified a significant indirect effect of DLPFC-rTMS on pain perception involving anticipatory CNV responses and anxiety ratings (indirect effect = −0.11, SE = 0.06, 95% CI [−0.29, −0.02], *p*_*FDR*_ = 0.014; Fig. 5B). Specifically, rTMS-related changes in anticipatory CNV and anxiety were jointly associated with subsequent changes in subjective pain perception.

These analyses revealed distinct stage-related indirect associations between expectancy-related neural responses and subsequent pain outcomes. Changes in contextual cue-evoked P2 were associated with subsequent laser-evoked N1 responses, whereas changes in anticipatory CNV and anxiety were associated with subjective pain ratings. It suggests that expectancy formation and maintenance make distinct contributions to subsequent pain processing.

## Discussion

The present study investigated how high-frequency DLPFC-rTMS influences pain through distinct expectancy-related processes operating across different stages of anticipation. Three main findings emerged. First, DLPFC-rTMS reduced both expected and perceived pain, and reductions in expected pain statistically mediated the effects of stimulation on pain perception, highlighting the importance of expectancy in prefrontal pain modulation. Second, DLPFC-rTMS differentially influenced expectancy formation and expectancy maintenance. During expectancy formation, real stimulation relatively increased contextual cue–evoked P2 amplitudes, whereas during expectancy maintenance it reduced anticipatory N1 and CNV responses. Third, these expectancy-related neural changes were linked to different pain outcomes. Mediation analyses suggested that expectancy formation was primarily associated with modulation of early nociceptive processing, whereas expectancy maintenance was more closely associated with changes in subjective pain experience. Together, these findings support the view that pain expectancy is a dynamic, multistage process rather than a unitary anticipatory state, with formation and maintenance making partly distinct contributions to subsequent pain processing. More broadly, the present results suggest that DLPFC-rTMS may influence pain by reshaping expectancy-related processing across multiple stages of anticipation.

The first important finding of the present study was that DLPFC-rTMS reduced subsequent pain intensity and unpleasantness, accompanied by a reduction in expected pain. Moreover, behavioral mediation analysis identified an indirect association between stimulation-related changes in expected pain and subsequent pain ratings, suggesting that modulation of expectancy may contribute to the analgesic effects of DLPFC-rTMS. This finding is consistent with evidence that pain perception is shaped not only by incoming nociceptive input but also by anticipatory processes [1], [4], [48]. Placebo and expectancy studies have demonstrated that anticipatory states can alter subsequent pain experience, with prefrontal engagement during anticipation associated with changes in pain-related neural responses and subjective pain ratings [10], [22], [23]. Similarly, previous neuromodulation studies have shown that DLPFC stimulation can influence pain anticipation and perception [27], [28], [29]. Our findings extend this literature by linking stimulation-related changes in expected pain to subsequent pain outcomes within a sham-controlled crossover design. However, DLPFC-rTMS did not produce significant overall changes in fear or anxiety ratings. This pattern suggests that the behavioral effects of stimulation were not accompanied by a generalized reduction across all measured expectancy-related emotional responses. Rather, changes in expected pain may represent one of several processes contributing to the observed analgesic effects, consistent with theoretical accounts of the DLPFC’s involvement in predictive processing and the modulation of subsequent perception [3], [25].

DLPFC-rTMS influenced the formation stage of expectancy, as reflected by changes in contextual cue-evoked P2 responses. Previous studies have linked the P2 component to the early allocation of attention toward task-relevant and predictive information [49], [50], [51], [52], [53], [54]. In expectancy paradigms, such attentional processing may facilitate the initial construction of predictive representations that guide subsequent perception. In the present study, DLPFC-rTMS attenuated the decline in contextual cue-evoked P2 amplitudes observed following sham stimulation, with this effect being more evident under the certain context. This pattern suggests that prefrontal stimulation relatively enhanced the early processing of predictive contextual information, potentially by modulating how attentional resources were allocated to behaviorally relevant cues during expectancy formation. Certain cues provide more reliable information about upcoming pain and may therefore support the formation of more stable expectancy representations [7], [33], [37]. No significant stimulation-related effects were observed for the later P3 component, suggesting that the influence of DLPFC-rTMS was more prominent during the earlier cue-processing period. Importantly, Importantly, mediation analysis identified a significant indirect association linking DLPFC-rTMS, contextual cue-evoked P2 responses, and subsequent laser-evoked N1 amplitudes. Whereas the vertex N2–P2 complex is commonly associated with the salience of incoming stimuli [55], [56], the laser-evoked N1 reflects relatively early cortical processing of nociceptive input, with contributions from somatosensory and insular regions [57], [58], [59], [60], [61]. Hence, DLPFC-rTMS-induced modulation of expectancy formation influences the early cortical processing of forthcoming nociceptive information.

Beyond expectancy formation, DLPFC-rTMS also influenced expectancy maintenance, as reflected by reduced anticipatory N1 and CNV responses. Previous studies have linked anticipatory N1 to early attentional orienting and vigilance toward upcoming events [7], [62], whereas CNV has been associated with sustained anticipation, temporal preparation, and the allocation of attentional resources during expectancy periods [62], [63]. Consistent with the involvement of attentional processes, Moscatelli et al. [64] reported that 10 Hz DLPFC-rTMS improved attentional performance, accompanied by changes in salivary Orexin-A levels, providing complementary evidence that high-frequency DLPFC stimulation can influence attention-related processing. In the present study, real stimulation reduced both anticipatory N1 and CNV amplitudes relative to sham stimulation, suggesting reduced neural engagement during expectancy maintenance, potentially involving changes in attentional orienting, sustained anticipation, and temporal preparation. Notably, these effects were observed across both certain and uncertain contexts, suggesting that DLPFC-rTMS modulated maintenance-related anticipatory processes shared across predictive contexts. Mediation analysis further identified a serial indirect effect linking DLPFC-rTMS to subsequent pain perception through anticipatory CNV responses and anxiety ratings. Although overall anxiety ratings were not significantly altered by stimulation, individual variations in these responses contributed to the identified indirect association. Thus, DLPFC-rTMS-induced modulation of expectancy maintenance may influence subsequent subjective pain experience through changes in anticipatory neural and affective processes.

Taken together, the present findings point to a stage-dependent pattern of DLPFC-rTMS effects across pain anticipation and experience. During expectancy formation, modulation of contextual cue-evoked P2 responses was more evident under certain contexts, whereas during pain experience, modulation of laser-evoked N1 responses was more pronounced under uncertainty. By contrast, anticipatory N1 and CNV responses during expectancy maintenance were robustly modulated across predictive contexts. This pattern suggests that the influence of predictive certainty on stimulation-related neural responses may vary across successive stages of pain processing. Reliable contextual cues provide more precise information about forthcoming pain and may therefore facilitate the formation of stable expectancy representations [7], [65], [66], whereas uncertainty leaves greater ambiguity regarding the upcoming outcome. Within predictive coding frameworks, the precision of prior expectations is thought to influence how predictive and sensory information are weighted during perception [3], [67], [68]. From a neuromodulation perspective, our findings suggest that the neural effects of DLPFC-rTMS are not expressed uniformly across the anticipation–pain sequence, but may vary according to predictive context. Under certain contexts, stimulation-related modulation was more evident during contextual cue processing, whereas under uncertainty it emerged more prominently in early pain-evoked responses. Hence, predictive context may influence when DLPFC-rTMS effects emerge across the anticipation–pain sequence.

The present findings have several theoretical, methodological, and potential translational implications. Theoretically, this study extends current accounts of expectancy-related pain modulation by treating anticipation as a dynamic process comprising distinguishable stages of expectancy formation and maintenance, rather than as a unitary anticipatory state [1], [7], [68]. DLPFC-rTMS modulated neural responses at both stages, and mediation analyses further showed that these stage-related changes were associated with different subsequent pain outcomes. Specifically, contextual cue-evoked P2 changes were linked to laser-evoked N1 responses, whereas anticipatory CNV and anxiety were associated with subjective pain ratings. These findings suggest that different stages of expectancy may contribute differently to subsequent pain processing, from early nociceptive responses to subjective pain experience [1], [3], [6], [68]. Methodologically, combining DLPFC-rTMS with EEG enabled us to track neural modulation across successive stages of anticipation and relate it to subsequent pain responses. From a translational perspective, identifying when and how prefrontal stimulation alters expectancy-related processing may help refine neuromodulatory approaches targeting maladaptive pain anticipation, particularly in conditions characterized by persistent expectations of pain or threat [15], [17], [18]. Whether these stage-related effects generalize to clinical populations and produce sustained benefits requires further investigation.

Several limitations should be acknowledged. First, the relatively small sample of healthy young adults may have limited sensitivity to modest stimulation effects and interactions and restricts the generalizability of the findings to clinical populations. Second, the present study examined only the immediate effects of a single rTMS session; whether repeated stimulation produces sustained changes in expectancy-related processing or clinically meaningful analgesic effects remains unknown. Third, the left DLPFC was targeted using the F3 position rather than individualized MRI-guided neuronavigation, which may have introduced interindividual variability in stimulation location. Finally, the ERP components examined here likely reflect multiple, partly overlapping processes, including expectancy, attention, and preparation, and the mediation analyses identify statistical indirect effects rather than definitive causal pathways. Future studies using larger and more diverse samples, individualized targeting, and repeated stimulation will help extend these findings toward clinical application.

In conclusion, the present study demonstrates that high-frequency DLPFC-rTMS reduces pain perception while modulating expectancy-related processes across distinct stages of anticipation. During expectancy formation, stimulation altered contextual cue–evoked P2 responses, whereas during expectancy maintenance it reduced anticipatory N1 and CNV activity. These stage-related changes were further linked to different subsequent pain outcomes, with contextual cue-evoked P2 associated with early laser-evoked N1 responses and anticipatory CNV and anxiety associated with subjective pain experience. Together, these findings move beyond a unitary view of pain expectancy and identify dynamic anticipatory processing as an important dimension of prefrontal pain modulation, with potential implications for refining noninvasive interventions targeting maladaptive pain anticipation.

## Author contributions

R.C.J.: Formal analysis; Writing – original draft; Writing – review & editing. Z.A.L.: Conceptualization; Data collection; Formal analysis. J.L.: Visualization; Writing – review & editing. Y.L.G.: Visualization; Writing – review & editing. G.H.W.: Writing – review & editing. X.Y.L.: Conceptualization; Formal analysis; Visualization; Writing – original draft; Writing – review & editing. W.W.P.: Conceptualization; Funding acquisition; Writing – original draft; Writing – review & editing.

## Declaration of competing interest

The authors declare that they have no known competing financial interests or personal relationships that could have appeared to influence the work reported in this paper.
