# Supplementary material for: Prefrontal rTMS modulates dynamic expectancy processes to shape subsequent pain experience
Source: Neurotherapeutics. 2026 Oct 28;24(1):e01107. doi: 10.1016/j.neurot.2026.e01107 (PMC13738117; doi:10.1016/j.neurot.2026.e01107)
Supplement: Supplementary file 1 [file mmc1.docx]

**Supplementary Materials**

**Table S1.** Average number of artifact-free trials retained per condition

| **Predictive context** | **Session** | **Cue-evoked responses** | **Laser-evoked responses** |
| --- | --- | --- | --- |
| **Certain** | Real–Pre | 19.97 ± 0.03 | 19.97 ± 0.03 |
|  | Real–Post | 20.00 ± 0.00 | 20.00 ± 0.00 |
|  | Sham–Pre | 20.00 ± 0.00 | 20.00 ± 0.00 |
|  | Sham–Post | 20.00 ± 0.00 | 19.97 ± 0.03 |
| **Uncertain** | Real–Pre | 39.97 ± 0.03 | 19.97 ± 0.03 |
|  | Real–Post | 40.00 ± 0.00 | 20.00 ± 0.00 |
|  | Sham–Pre | 40.00 ± 0.00 | 19.97 ± 0.03 |
|  | Sham–Post | 40.00 ± 0.00 | 20.00 ± 0.00 |

**Note.** Values are presented as mean ± SEM and represent artifact-free trials retained after EEG preprocessing. In the uncertain condition, 40 contextual/anticipatory cue trials were presented, of which 20 were followed by laser stimulation; therefore, the maximum number of cue-evoked and laser-evoked trials was 40 and 20, respectively. Trial retention exceeded 95% across all conditions.

**Baseline comparability between the real- and sham-rTMS sessions**

Pre-stimulation values were compared between the real- and sham-rTMS sessions using paired-samples t-tests after averaging across predictive contexts (Supplementary Table S2). Baseline values were generally comparable between sessions, except for anticipatory N1 amplitude (*t_32_* = −2.21, *p* = 0.034).

To assess whether this baseline difference influenced the stimulation-related N1 effect, we conducted a baseline-adjusted linear mixed-effects analysis using post-stimulation N1 amplitude as the dependent variable and pre-stimulation amplitude as a covariate. The main effect of rTMS Type remained significant (*F* = 5.68, *p* = 0.019), whereas the rTMS Type × Predictive Context interaction was not significant (*F* = 0.60, *p* = 0.441), indicating that the stimulation-related N1 effect remained evident after baseline adjustment.

**Table S2.** Baseline comparison of behavioral and electrophysiological outcomes between the real- and sham-rTMS sessions

| **Outcome** | **Real-rTMS session** | **Sham-rTMS session** | ***t_32_*** |
| --- | --- | --- | --- |
| ***Behavioral responses*** | | | |
| Expected pain | 5.48 ± 0.29 | 5.52 ± 0.28 | −0.23 |
| Fear | 4.70 ± 0.35 | 4.95 ± 0.41 | −1.07 |
| Anxiety | 4.38 ± 0.40 | 4.69 ± 0.44 | −1.20 |
| Pain intensity | 5.04 ± 0.30 | 5.23 ± 0.28 | −0.99 |
| Unpleasantness | 3.50 ± 0.35 | 3.58 ± 0.39 | −0.42 |
| ***Expectancy formation*** | | | |
| Contextual P2 | 4.03 ± 0.50 | 4.84 ± 0.47 | −1.81 |
| Contextual P3 | 0.91 ± 0.52 | 1.33 ± 0.52 | −0.76 |
| ***Expectancy maintenance*** | | | |
| Anticipatory N1 | −1.38 ± 0.32 | −0.68 ± 0.29 | **−2.21*** |
| CNV | −1.95 ± 0.52 | −1.22 ± 0.48 | −1.04 |
| ***Pain experience*** | | | |
| N2–P2 complex | 39.83 ± 2.71 | 42.74 ± 3.05 | −1.22 |
| Laser-evoked N1 | −4.26 ± 1.01 | −4.10 ± 1.23 | −0.19 |

**Notes:** Values are presented as mean ± SEM and represent pre-stimulation measurements averaged across certain and uncertain predictive contexts. Baseline values were compared using paired-samples *t*-tests (*n* = 33). Significant differences are shown in bold. **p* < 0.05 (uncorrected).

**Repeated-measures analyses of behavioral and electrophysiological outcomes**

To complement the primary change-score analyses, the original pre- and post-stimulation measurements were analyzed using three-way repeated-measures ANOVAs with rTMS Type (real vs. sham), Predictive Context (certain vs. uncertain), and Time (pre vs. post) as within-subject factors. The rTMS Type × Time interaction corresponded to the rTMS Type effect in the change-score analyses, whereas the rTMS Type × Predictive Context × Time interaction corresponded to the rTMS Type × Predictive Context interaction. These corresponding effects reproduced the pattern observed in the primary analyses, and the full repeated-measures models did not alter the interpretation of the findings. Complete results are presented in Supplementary Table S3.

**Table S3.** Three-way repeated-measures analyses of behavioral and electrophysiological outcomes

| **Outcome** | **rTMS Type** | **Context** | **Time** | **rTMS Type × Time** | **Context × Time** | **rTMS Type × Context** | **rTMS Type × Context × Time** |
| --- | --- | --- | --- | --- | --- | --- | --- |
| ***Behavioral responses*** | | | | | | | |
| Expected pain | 2.01 | **5.91*** | **11.75**** | **5.46*** | 0.31 | 0.20 | 0.02 |
| Fear | 2.96 | 0.31 | **29.37***** | 2.76 | 0.002 | 0.55 | 0.04 |
| Anxiety | 2.61 | 0.09 | **13.80***** | 1.36 | <0.001 | 0.39 | 0.12 |
| Pain intensity | 2.62 | 2.53 | **38.58***** | **4.22*** | 1.34 | 0.03 | 0.03 |
| Unpleasantness | 2.67 | 0.73 | **16.04***** | **5.46*** | 0.30 | 2.21 | 0.42 |
| ***Expectancy formation*** | | | | | | | |
| Contextual P2 | 0.11 | **20.97***** | **6.04*** | **8.40**** | 1.33 | 0.01 | **5.22*** |
| Contextual P3 | 0.40 | 1.96 | **8.16**** | 0.18 | 0.01 | 0.05 | 0.26 |
| ***Expectancy maintenance*** | | | | | | | |
| Anticipatory N1 | 0.11 | 0.63 | 1.70 | **9.60**** | 0.38 | 1.03 | 1.74 |
| CNV | 0.04 | 3.79 | 0.09 | **6.94*** | 0.81 | 0.14 | 0.56 |
| ***Pain experience*** | | | | | | | |
| N2–P2 complex | 1.86 | **7.74**** | **49.22***** | 0.06 | 1.16 | 0.30 | 0.37 |
| Laser-evoked N1 | 0.04 | 0.97 | 0.94 | 1.20 | 0.71 | **8.72**** | **4.69*** |

**Notes:** Values are *F_(1, 32)_* statistics from three-way repeated-measures ANOVAs with rTMS Type (real vs. sham), Predictive Context (certain vs. uncertain), and Time (pre vs. post) as within-subject factors. The rTMS Type × Time interaction corresponds to the rTMS Type effect in the Post–Pre change-score analyses, whereas the rTMS Type × Predictive Context × Time interaction corresponds to the rTMS Type × Predictive Context effect. Significant effects are shown in bold. Asterisks indicate uncorrected statistical significance: **p* < 0.05; ***p* < 0.01; ****p* < 0.001.

**Effects of stimulation sequence on behavioral and electrophysiological outcomes**

To examine whether stimulation sequence influenced the observed effects, we conducted three-way mixed-design ANOVAs on Post–Pre change scores, with rTMS Type (real vs. sham) and Predictive Context (certain vs. uncertain) as within-subject factors and Stimulation Sequence (real-first vs. sham-first) as a between-subject factor. Complete results are presented in Supplementary Table S4.

The overall pattern of stimulation-related effects was largely preserved after accounting for stimulation sequence. No significant rTMS Type × Stimulation Sequence interactions were observed for any outcome, indicating no statistically significant evidence that the overall rTMS effects differed between the real-first and sham-first groups. The main rTMS effects on expected pain, pain unpleasantness, contextual P2, anticipatory N1, and CNV remained significant, whereas the effect on pain intensity showed a similar pattern but did not reach significance (*F_1, 31_* = 4.06, *p* = 0.053, η_p_² = 0.12).

One exception was a significant rTMS Type × Predictive Context × Stimulation Sequence interaction for expected pain (*F_1, 31_* = 6.10, *p* = 0.019, η_p_² = 0.17; Supplementary Fig. S1), indicating that the context-dependent pattern of expected-pain changes differed between stimulation sequences. We therefore interpret context-specific effects on expected pain with appropriate caution.

**Figure S1. Changes in expected pain as a function of stimulation sequence and predictive context.** Post–Pre changes in expected pain ratings are shown separately for the real-first and sham-first sequence groups under certain and uncertain predictive contexts. Bars represent mean ± SEM; negative values indicate reductions in expected pain. Asterisks indicate significant real-versus-sham differences within the corresponding sequence and context (**p* < 0.05).

**Table S4.** Stimulation-sequence effects on behavioral and electrophysiological outcomes

| **Outcome** | **rTMS Type** | **Context** | **Sequence** | **rTMS Type × Sequence** | **Context × Sequence** | **rTMS Type × Context** | **rTMS Type × Context × Sequence** |
| --- | --- | --- | --- | --- | --- | --- | --- |
| ***Behavioral responses*** | | | | | | | |
| Expected pain | **5.54*** | 0.29 | 1.45 | 0.02 | 0.29 | 0.05 | **6.10*** |
| Fear | 2.67 | 0.001 | 0.24 | <0.001 | 1.12 | 0.05 | 0.40 |
| Anxiety | 1.09 | 0.01 | 0.02 | 0.11 | 1.07 | 0.17 | 2.24 |
| Pain intensity | 4.06 | 1.32 | 2.13 | 0.46 | 0.12 | 0.02 | 0.52 |
| Unpleasantness | **5.26*** | 0.33 | 0.33 | 0.29 | 0.90 | 0.40 | 0.08 |
| ***Expectancy formation*** | | | | | | | |
| Contextual P2 | **8.61**** | 1.26 | 0.01 | 1.12 | 0.99 | **5.08*** | 0.04 |
| Contextual P3 | 0.23 | 0.01 | 1.32 | 2.55 | 0.69 | 0.26 | 0.08 |
| ***Expectancy maintenance*** | | | | | | | |
| Anticipatory N1 | **9.39**** | 0.34 | 1.48 | 0.13 | 1.72 | 1.73 | 0.21 |
| CNV | **6.81*** | 0.76 | <0.001 | 0.14 | 0.20 | 0.60 | 0.87 |
| ***Pain experience*** | | | | | | | |
| N2–P2 complex | 0.05 | 1.09 | 1.70 | 0.93 | 0.59 | 0.35 | 0.05 |
| Laser-evoked N1 | 1.20 | 0.68 | 0.03 | 0.21 | <0.001 | **4.52*** | 0.05 |

Notes: Values are *F_(1, 31)_* statistics from three-way mixed-design ANOVAs conducted on Post–Pre change scores, with rTMS Type (real vs. sham) and Predictive Context (certain vs. uncertain) as within-subject factors and Stimulation Sequence (real-first vs. sham-first) as a between-subject factor. Significant effects are shown in bold. Asterisks indicate uncorrected statistical significance: *: *p* < 0.05; **: *p* < 0.01.
